# The complete chloroplast genome sequence of *Dendrobium zhenghuoense* (Orchidaceae)

**DOI:** 10.1080/23802359.2019.1669086

**Published:** 2019-10-03

**Authors:** Yuan-Zhen Huang, Li-bin Zhuang, Jun-Wen Zhai, Wen-Jun Lin

**Affiliations:** a Fujian Agriculture and Forestry University, Fuzhou, China;; b Fuzhou Botanical Garden, Fuzhou, China

**Keywords:** *Dendrobium zhenghuoense*, chloroplast genome, phylogeny

## Abstract

*Dendrobium zhenghuoense* is very distinctive among Orchidaceae species with yellowish-green flowers and short peduncle. In this study, we reported the complete chloroplast genome of *D. zhenghuoense.* Present study suggests that genomic information of *D. zhenghuoense* would have special importance in conservation and molecular phylogenetic studies on Orchidaceae. The circle genome of *D. zhenghuoense* was 148,431 bp in length with 37.0% GC content and a large single-copy (LSC) region of 84,167 bp, a small single-copy (SSC) region of 12,588 bp, which were separated by a pair of inverted repeat (IR) region of 25,838 bp. The genome contained 130 genes, including 75 protein-coding genes, 38 tRNA genes and 8 rRNA genes. The maximum likelihood phylogenetic analysis indicated that *D. zhenghuoense* was the sister to the rest 11 species of *Dendrobium* tested.

*Dendrobium* Swartz is one of the largest genera in Orchidaceae (Pridgeon et al. 2014) and comprises approximately 1450 species. Approximately 80 species of *Dendrobium* are found in China, of which 18 species are endemic (Zhu et al. 2009; Liu and Chen 2011; Xu et al. 2014; Deng et al. 2016). *D. zhenghuoense* was first discovered in Zhenghe County, Fujian, China (Chen et al. 2016). It differs greatly in having yellowish green flowers, shorter peduncle, longer lip and mentum from *Dendrobium luoi*. In this study, we firstly reported the complete chloroplast genome of *D. zhenghuoense* based on Illumina sequencing technology, which will be contributed to the further studies of the whole genus. We also constructed a phylogeny to confirm its relationship and compared this sequence with those from other 14 species of Orchidaceae.

The fresh leaf was sampled from Zhenghe where the model species originated. And the specimens were deposited in the herbarium of Fujian Agriculture and Forestry University (specimen code: FAFU Chen SP, 0220). Total DNA was extracted from fresh leaves using a modified CTAB method (Doyle and Doyle 1987) and sequenced by the BGISEQ500 sequencing platform. We performed the assembling by GetOrganelle pipe-line (https://github.com/Kinggerm/GetOrganelle), it can get the plastid-like reads, and the reads were viewed and edited by Bandage (Cao et al. 2019). Then using GENEIOUS R10 (Biomatters Ltd., Auckland, New Zealand) (Kearse et al. 2012) to correct the annotation by compared with the sequence of *Dendrobium shixingense* (GenBank accession No. LC348722). Finally, we obtained an accurate chloroplast genome of and submitted *D. zhenghuoense* the whole genome to GenBank (MN164627).

The circular genome of *D. zhenghuoense* was 148,431 bp in length and comprised a large single-copy (LSC) region of 84,167 bp, a small single-copy (SSC) region of 12,588 bp, and two inverted repeat (IRa and IRb) regions of 25,838 bp. The genome contained 130 genes, including 75 protein-coding genes, 38 tRNA genes and 8 rRNA genes. Overall GC content of the whole genome is 37.0%, while the corresponding values of the LSC, SSC, and IR regions are 34.4%, 29.2%, and 43.2%, respectively.

To further investigate its phylogenetic position, the sequences of *D. zhenghuoense* and other 13 species (downloaded from NCBI GenBank) were firstly aligned using pipeline (Bi et al. 2018). Then, RAxML-HPC Black-Box version 8.1.24 (Stamatakis 2014) was used to construct a maximum likelihood tree with *Cymbidium sinense* and *Erycina pusilla* as outgroups. The branch support was computed with 1000 bootstrap replicates. In the study, the maximum likelihood phylogenetic analysis indicated that *D. zhenghuoense* was sister to the other 11 species of *Dendrobium* (Figure 1).

**Figure 1.**
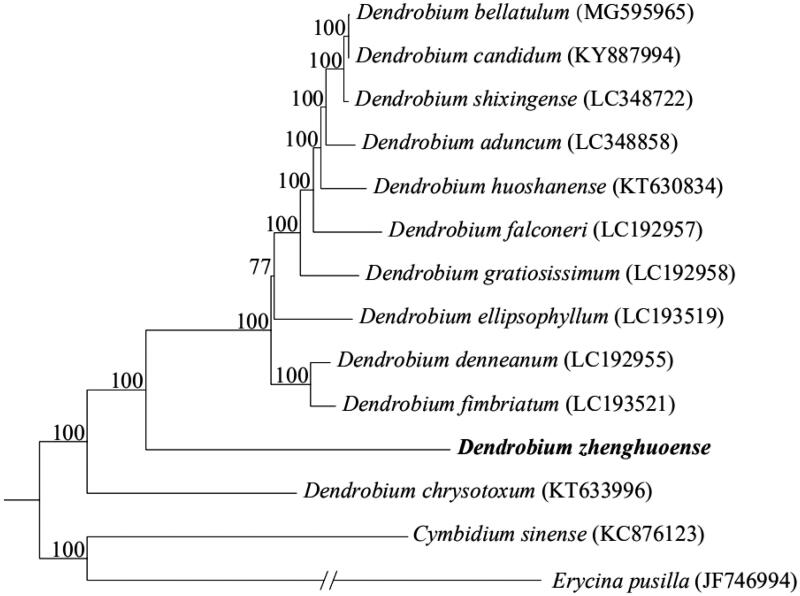
Maximum likelihood tree based on the complete chloroplast genome sequences of 14 species from the Orchidaceae with *Cymbidium sinense* (Orchidaceae) and *Erycina pusilla* (Orchidaceae) as outgroup. Shown next to the nodes are bootstrap support values based on 1000 replicates.
